# Supplementary material for: Synthesis of Calcium Silicate Hydrate from Coal Gangue for Cr(VI) and Cu(II) Removal from Aqueous Solution
Source: Molecules. 2021 Oct 14;26(20):6192. doi: 10.3390/molecules26206192 (PMC8537422; doi:10.3390/molecules26206192)
Supplement: Supplementary file 1 [file molecules-26-06192-s001.zip › molecules-1388801-supplementary.pdf]

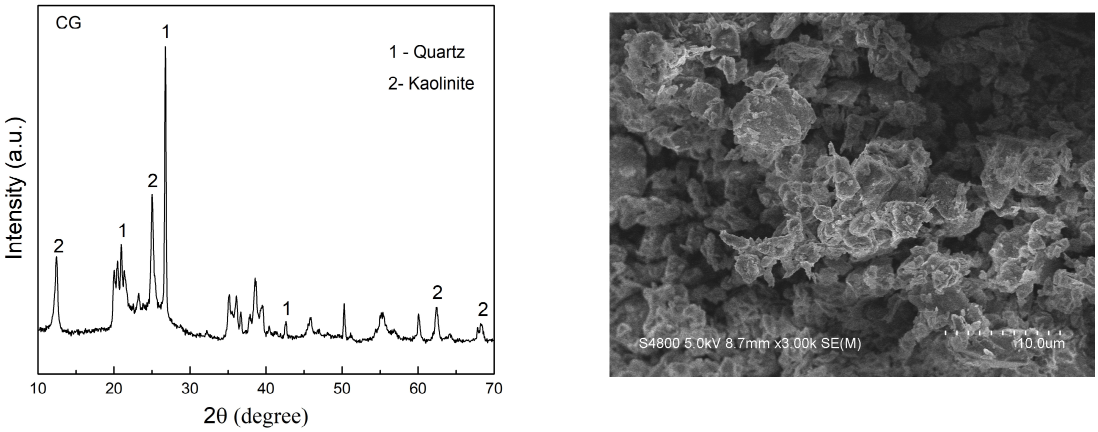

(a)

(b)

Figure S1. The XRD patterns (a) and SEM observation (b) of coal gangue.

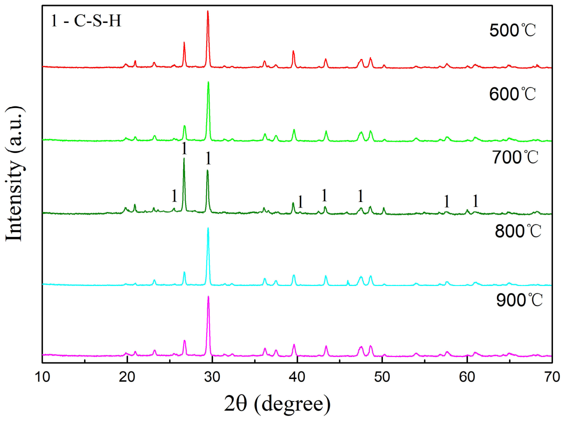

(a)

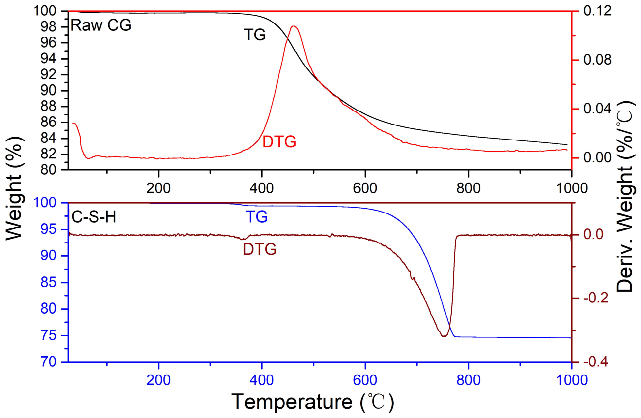

(b)

Figure S2. The XRD patterns (a) and TG-DTG curves (b) of the synthesized samples at various calcination temperatures.

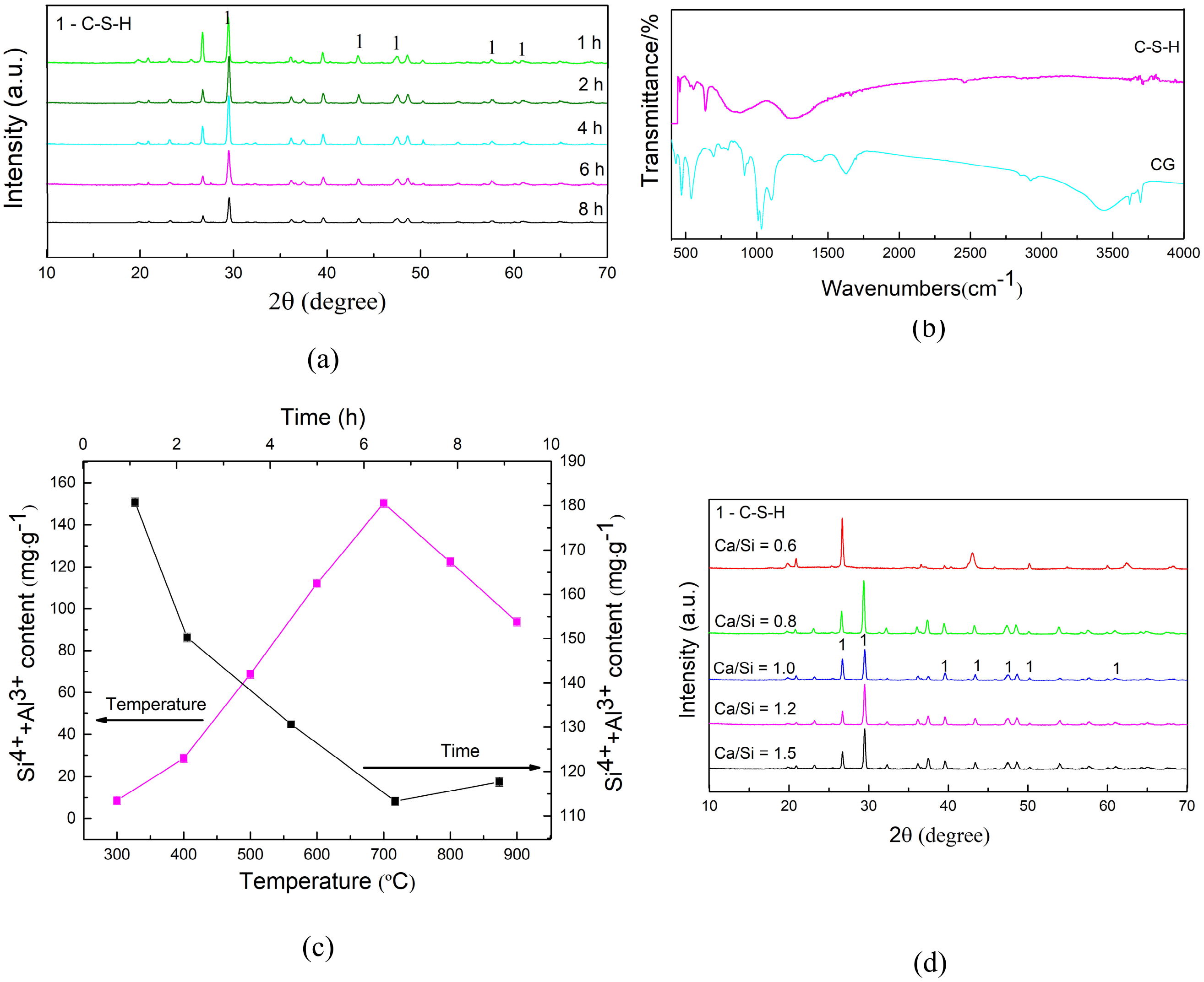

Figure S3. The XRD patterns of the synthesized samples at various calcination time (a), FTIR spectra (b) of coal gangue and C-S-H at 700°C and 1 h, total dissolution amount of Si^{4+} and Al^{3+} from C-S-H in NaOH solution at 700°C and 1 h (c), XRD patterns of the synthesized samples at various Ca/Si ratio (d).

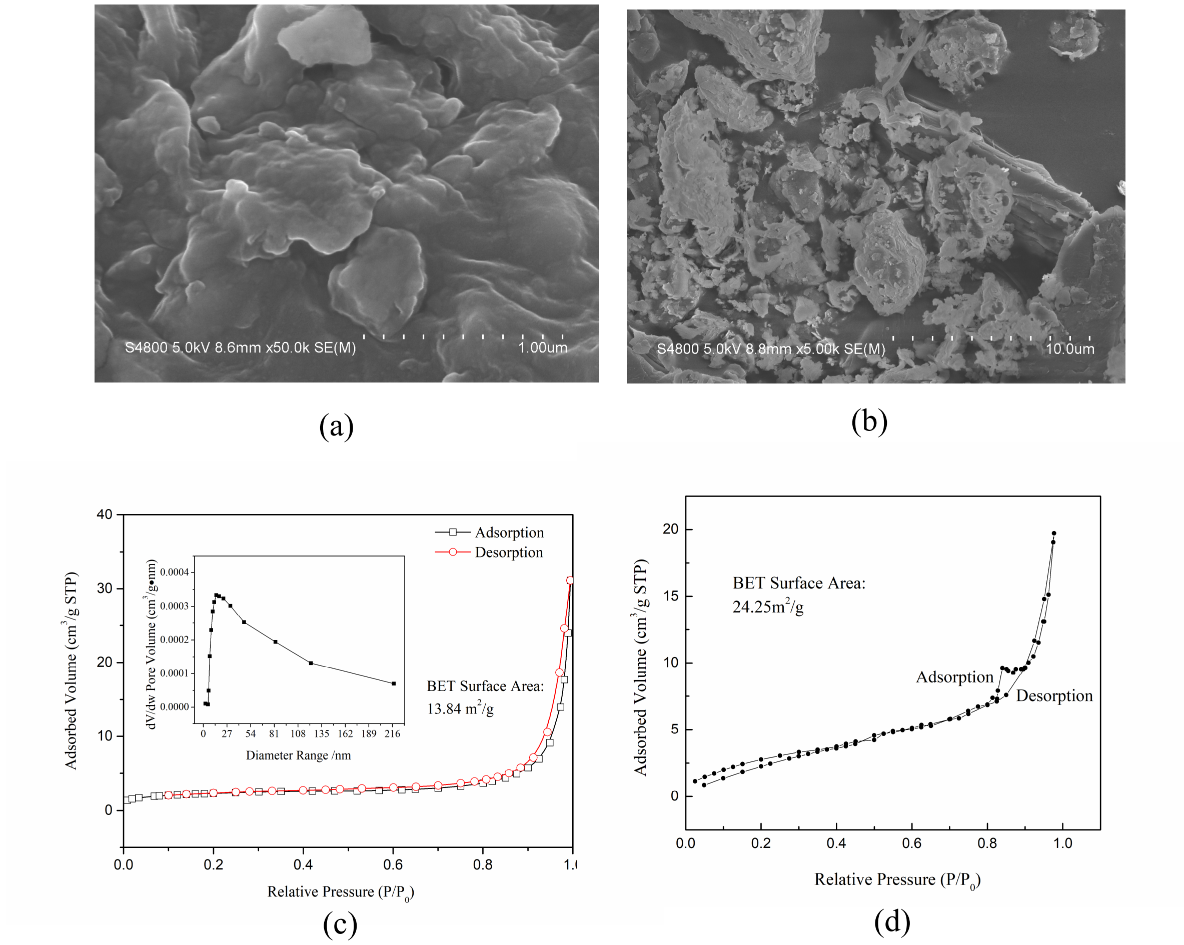

Figure S4. SEM observation of C-S-H (a and b) and BET surface area and pore volume of coal gangue (c) and C-S-H (d).

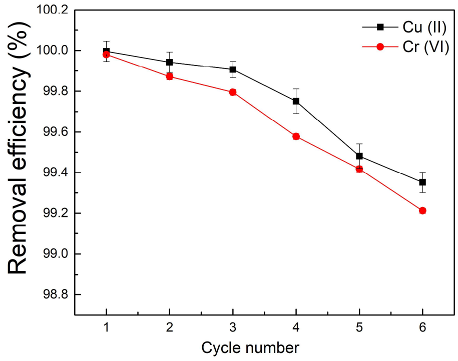

Figure S5. Effect of cycle number for Cu(II) and Cr(VI) adsorption onto C-S-H.

Table S1

The adsorption thermodynamics parameters for Cu(II) and Cr(VI) adsorption onto C-S-H.

Adsorbate	K_0	ΔG^0 ($kJ \cdot mol^{-1}$)	ΔH^0 ($kJ \cdot mol^{-1}$)	ΔS^0 ($kJ \cdot mol^{-1} \cdot K^{-1}$)
Cu(II)	1249.90	-17.67	13.89	7.32
	277.68	-14.17		
	178.68	-13.28		
	104.07	-12.17		
	45.35	-10.08		
Cr(VI)	2499.90	-19.38	33.39	14.88
	312.40	-14.47		
	124.90	-12.36		
	78.02	-11.41		
	62.4	-10.93		
